# MP3: Medical Software for Processing Multi-Parametric Images Pipelines

**DOI:** 10.3389/fninf.2020.594799

**Published:** 2020-11-16

**Authors:** Clément Brossard, Olivier Montigon, Fabien Boux, Aurélien Delphin, Thomas Christen, Emmanuel L. Barbier, Benjamin Lemasson

**Affiliations:** ^1^University of Grenoble Alpes, INSERM, U1216, Grenoble Institut des Neurosciences (GIN), Grenoble, France; ^2^MoGlimaging Network, HTE Program of the French Cancer Plan, Toulouse, France; ^3^University of Grenoble Alpes, Inria, CNRS, G-INP, Grenoble, France

**Keywords:** software, medical images, image processing, pipelines, database, MRI, CT

## Abstract

This article presents an open source software able to convert, display, and process medical images. It differentiates itself from the existing software by its ability to design complex processing pipelines and to wisely execute them on a large databases. An MP3 pipeline can contain unlimited homemade or ready-made processes and can be carried out with a parallel execution system. As a viewer, MP3 allows display of up to four images together and to draw Regions Of Interest (ROI). Two applications showing the strengths of the software are presented as examples: a preclinical study involving Magnetic Resonance Imaging (MRI) data and a clinical one involving Computed Tomography (CT) images. MP3 is downloadable at https://github.com/nifm-gin/MP3.

## 1. Introduction

Researchers in medical imaging now have access to large amounts of data via open source databases [The Cancer Imaging Archive (TCIA; Dataset, 2020b), Center Traumatic Brain Injury (CTBI; Dataset, 2020a)]. In parallel of the quantity of images available, the complexity of the medical images post-processing is increasing. Where data scientists applied a single process to their images to obtain valuable results (Lemasson et al., 2016), state of the art analysis requires the execution of very complex interdependent processes called *pipelines* involving the execution of many operations, referred to as *modules*, on large databases (Funck et al., 2018). Among the modules used (e.g., bias removal, brain extraction, or image registration) some of them are part of toolboxes well-recognized by the community [such as SPM (Software, 2020) or FSL (Jenkinson et al., 2012) for the neuroimaging community] whereas others are home-made. How to reconcile flexibility, adaptive, speed, performance, and reproducibility of a post-processing? Several software have been recently developed to solve this issue. One can divide them in two classes: specific and generic software. Specific one, are built to process a specific type of data, for instance a modality [e.g., Positron Emission Tomography (PET), Magnetic Resonance Imaging (MRI),...], an organ imaged (e.g., liver, brain,...), or even a format [e.g., Digital imaging and communications in medicine (DICOM), Neuroimaging Informatics Technology Initiative (NIfTI),...] using a predefined pipeline. On the other hand, generic software, aimed to apply different processes on several types of data. Most of the available software can be gathered in the first category. This includes BrainCAT (Marques et al., 2013) which generates diffusion tensor images (DTI) from MRI diffusion weighted images, MRtrix (Tournier et al., 2019), which allows visualization and specific processes on diffusion MRI data, Pydpiper (Friedel et al., 2014) which offers registration algorithms, or FuNP (Park et al., 2019) which gathers some of the most widely used functions available for MRI data in a single post-processing pipeline. In those software, either the type of input data or the processing applied is unalterable. The second category contains software that can apply many different processes on a large type of data. To our knowledge, only few software available in free access belong to this category. One can quote Vaa3D (Peng et al., 2010), able to handle large dataset of images converted in Tagged Image File Format (TIFF) in a few seconds, to display them in 3D, and to define and execute homemade pipelines. One can also refer to GraphMIC (Zehner et al., 2015), which provides a node based interface for famous image processing libraries and allows to link the modules of these libraries to create complex image processing pipelines without programming, simply by connecting existing modules. This powerful software makes the design of complex pipeline user-friendly as well as the integration of home-made modules. Those powerful software lack of functionalities that we believe will become essential to develop new imaging biomarkers in order to handle and process large heterogeneous databases. Instead of processing one file or subject at a time, one will need a tool able to handle large cohorts, containing heterogeneous (multi-format) and multi-parametric data, and to process them with specific complex pipelines and with respect to the intra-subject time dependencies. To answer these limitations, we propose an open source software called “*Medical software for Processing multi-Parametric images Pipelines (MP3)*” (Software, 2019). This software therefore aims to facilitate the design of complex image analysis pipelines using existing or home-made modules as well as their execution on large heterogeneous databases.

## 2. Method

### 2.1. Overview

**Figure 1:**
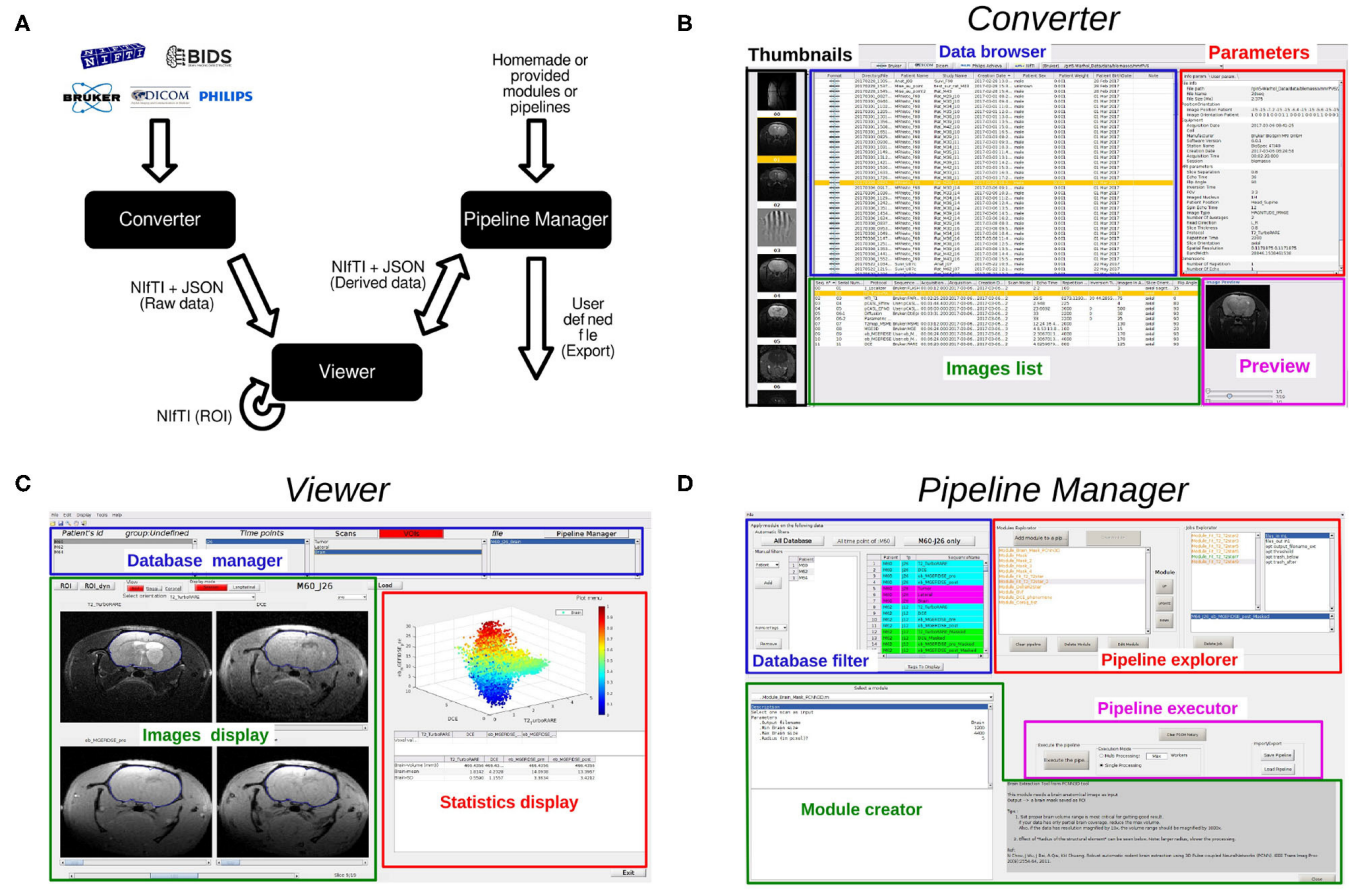
**(A)**: Architecture overview and data flow, **(B)**: Converter, **(C)**: Viewer, **(D)**: Pipeline Manager.

MP3, a MATLAB (Software, 2017) toolbox, intends to assist an end-to-end research study, from the loading and the converting of raw images to the statistical analysis through the creation of a database containing metadata and the design and execution of complex analysis pipelines. MP3 is composed of three linked graphical user interfaces (GUI) that stands as its backbone: the converter, the viewer, and the pipeline manager (Figure 1A). Briefly, those GUI enable the conversion, display, and processing of different medical image formats and architectures (Bruker, DICOM, PAR/REC, NIfTI, BIDS; Gorgolewski et al., 2016). The imported data is summarized in a database able to be homogenized, filtered, or improved with metadata such as the name of the patient or the day of the acquisition. The viewer can display up to four 5D images simultaneously and to draw ROI. Eventually, a graphical interface called Pipeline Manager creates, manages, and executes complex pipelines using editable modules. A pipeline execution system named PSOM (v2.2.2) (Bellec et al., 2012) allows to judiciously parallelize the execution of the modules. In this section, we detail the aims and functionalities specific to each of these GUI and present the concept of an MP3 project and its architecture. Detailed information as well as videos presenting latest developments of MP3 are available online (Github: https://github.com/nifm-gin/MP3, YouTube: https://www.youtube.com/playlist?list=PL-Tj6Wc9aE9x7i6s-RLetvNE0isnEsFm7).

### 2.2. Data Import and Conversion

MP3 is built on the NIfTI format (Cox et al., 2004), which contains the imaged volumes as a matrix (up to seven dimensions), and a short header that stores information about the image as its type or its size. This header also contains a transformation matrix that describes the position of the volume in a conventional space which is very useful to co-register two NIfTI volumes without modifying the image. In order to store metadata of each image acquisition that may be essential for some post-processing (echo time, repetition time, etc.), a consortium led by France Life Imaging defined conventional metadata and a way to write them in a JSON file (Kain et al., 2020). A pair of NIfTI/JSON files entirely describes a medical image. To allow the use of classical formats of medical images, we developed a converter able to transform Bruker, PAR/REC, DICOM, and NIfTI files to a NIfTI plus JSON format. It is also possible to import data organized in the Brain Imaging Data Structure (BIDS) convention. Each user can also customize the metadata to be collected from the raw data (Bruker, DICOM, etc.) that will be stored in the JSON file by editing the provided YAML file. Full details about the customization of the image conversion is available online (https://populse.github.io/mri_conv/Home/index.html). The converter interface (Figure 1B) can be launched from the main MP3 window. To start a new project, one defines a folder to store the project. Then, thanks to the different parts of the converter (data browser, parameters, images list, etc...), one can easily navigate through complex and often human unreadable medical image architecture. When the desired images are found, they will be automatically converted to NIfTI/JSON and metadata such as timepoints and subjects names may be modified. Then, back to the viewer GUI, a database has been created and will be used to manage the project data (Video 1: https://www.youtube.com/watch?v=ebofxMSquFs&list=PL-Tj6Wc9aE9x7i6s-RLetvNE0isnEsFm7).

### 2.3. Project Architecture

On the hard drive, the main directory of each project, sorted in multiple sub-folders, contains all the data needed to open the project via MP3. We defined several types of files, among which *Scans*, classical medical images, described as a NIfTI and a JSON file, and *ROI*, described as a NIfTI file (containing a binary matrix). Each imported scan file is stored in the “Raw_data” sub-folder, while the processed files, written after a pipeline execution, are saved in the “Derived_data” sub-folder and the ROI in “ROI_data.” Other sub-folders can be part of the project, such as for instance “Tmp,” where lie temporary files, or “Saved_Pipelines,” that stores the .mat files that summarize the designed pipelines (see section 2.5). To manage these sub-folders, files, and associated metadata, MP3 relies on a database containing, for each entry, information referred to as *tags*, as its Type-Tag (Scan or ROI), Subject-Tag, Timepoint-Tag, or Group-Tag associated, as well as its Path-Tag or Filename-Tag. Since MP3 is developed on MATLAB, we decided not to use a real database, as we could have developed in SQL language, but to use a MATLAB *table* object. The power of these variables relies on their ability to be quickly and easily filtered, which is a key operation in MP3. Each entry is linked to the corresponding files through this table. It is therefore this database that undergoes operations as renaming, sorting, or filtering to select part of our available data or to homogenize the database to make it more consistent. Since a project is completely described by its database, MP3 offers the possibility of easily transferring a whole project to another user. To save space and to easily share any project, MP3 handles the compressed NIfTI format *.nii.gz*.

### 2.4. Viewer

The Viewer is a MATLAB GUI that is divided into three parts (Figure 1C): a *database manager*, to display and manage the database, the *image display*, that displays up to four 5D images from the database and supports the drawing of ROIs, and the *statistics display*, which displays scatter plots, curves, histograms, or first order statistics, like mean or standard deviation within a ROI (Video 2: https://www.youtube.com/watch?v=X26RV7VmXTA&list=PL-Tj6Wc9aE9x7i6s-RLetvNE0isnEsFm7).

#### 2.4.1. Database Manager

The database (section 2.3) is presented as four listboxes each displaying a tag (Subject-Tag, Timepoint-Tag, Sequence_Name-Tag, and Filename-Tag) and working as filters that reduce the database. Thereby, when the Subject-Tag list displays all the different subjects of the database, selecting one of them filters the database and reduces the files displayed in the other lists. This way of presenting the database is inspired by the BIDS architecture and is a simple and efficient way to quickly access a specific scan. One can switch from the Scans list to the ROIs list by just a click on the specific button above the Sequence_Name-Tag list. These lists allow renaming the value of a tag, copying, or deleting an entry in the database thanks to specific right-click menus associated with each listbox. To compute these operations on a large number of entries, a sub-menu of the Edit menu offers some “Delete from database” or “Rename from database” features.

#### 2.4.2. Image Display

One can load and display up to four 5D scans simultaneously. One of the main advantages of the NIfTI files is their transformation matrix stored in the header (section 2.2). This matrix locates the center of the scanner and its geometry. It is therefore possible, thanks to a light interpolation provided by functions of the SPM toolbox (Software, 2020), to display different orientations and different resolutions. This ability is used to open the selected scans in a selected referential. One can change the selected referential thanks to a popup menu displayed on the top of the figures. Open different scans in the same referential is particularly useful when displaying two scans acquired with a different field of view or a different voxel size. Three push buttons above the images can be used to change the referential orientation among axial, sagittal, or coronal. On a displayed image, one can use some classic features such as zoom and pan the image or vary its contrast to see hidden patterns (right-click on the mouse). One can also set the same contrast to all displayed images, and then quantitatively compare several images values (middle-click on the mouse). The viewer also offers a graphical tool able to create a contour that defines a ROI. This contour can be manually or automatically drawn thanks to an algorithm based on an active contour growth (Wang et al., 2009). ROIs are automatically stored as NIfTI files and can then be displayed on any other scan, regardless of its orientation or geometry.

#### 2.4.3. Statistics Display

This part of the viewer is aimed at displaying quantitative information about the scans or ROIs loaded. It incorporates a sub-window to display graphics and a table for numeric values. The first metric is the values of the voxels. When the mouse pointer flies over an image, the value of the current voxel on each loaded scan is displayed on the table, which gives a quick overview of the quantitative values of each image. Another MP3 feature is its ability to study the temporal evolution of a four dimensional scan. Indeed, a click on a 4D voxel displays its values along the fourth dimension, which is particularly useful to study MRI perfusion or functional MRI. When an ROI is loaded, mean and standard deviation within the ROI for each loaded scan are displayed in a table, in addition to its volume in mm^3^. When only one scan is loaded, the figure above that table hosts a histogram and when several scans are displayed, this histogram is replaced by a scatter plot, which represents the pixel values of one scan vs. the others. Since the MP3 project is open-source, one can easily display any simple feature or statistics by editing some of the main functions.

#### 2.4.4. Longitudinal Follow-Up View Mode

Another way to analyze images from the database is to switch the display mode of the viewer from *session* to *longitudinal*. This mode allows comparison of scans taken at different times for a single patient. One can then easily check a co-registration, and conduct volumetric analyses.

### 2.5. Pipeline Manager

Since the basic features (section 2.4.3) are not enough to process complex analyses and are limited to the analysis of one subject we developed a third graphical user interface called the *Pipeline Manager* (Figure 1D). The pipeline manager allows the creation, editing, saving, and execution of complex pipelines on any file of a project. Moreover, it integrates fast and reproducible computation processes (mutli CPU) and history handling. This section exposes the philosophy of the pipeline manager and the main functionalities of this GUI (Video 3: https://www.youtube.com/watch?v=QOULzRsrzzg&list=PL-Tj6Wc9aE9x7i6s-RLetvNE0isnEsFm7).

#### 2.5.1. Principal of the Pipeline Manager

Thanks to our structured project—data conversion (section 2.2) and database (section 2.3), the pipeline manager is prepared to manipulate our data. The pipeline manager allows iterations of a given process over the whole database (or a sub-part of the database). For instance, we are able to create a process that computes an image *B* from one specific image *A* and to apply it on each image *A* of the database. This process is called *pipeline*, because it can be a simple computation or a complex sequence of independent steps.

#### 2.5.2. Pipeline Definition

**Figure 2:**
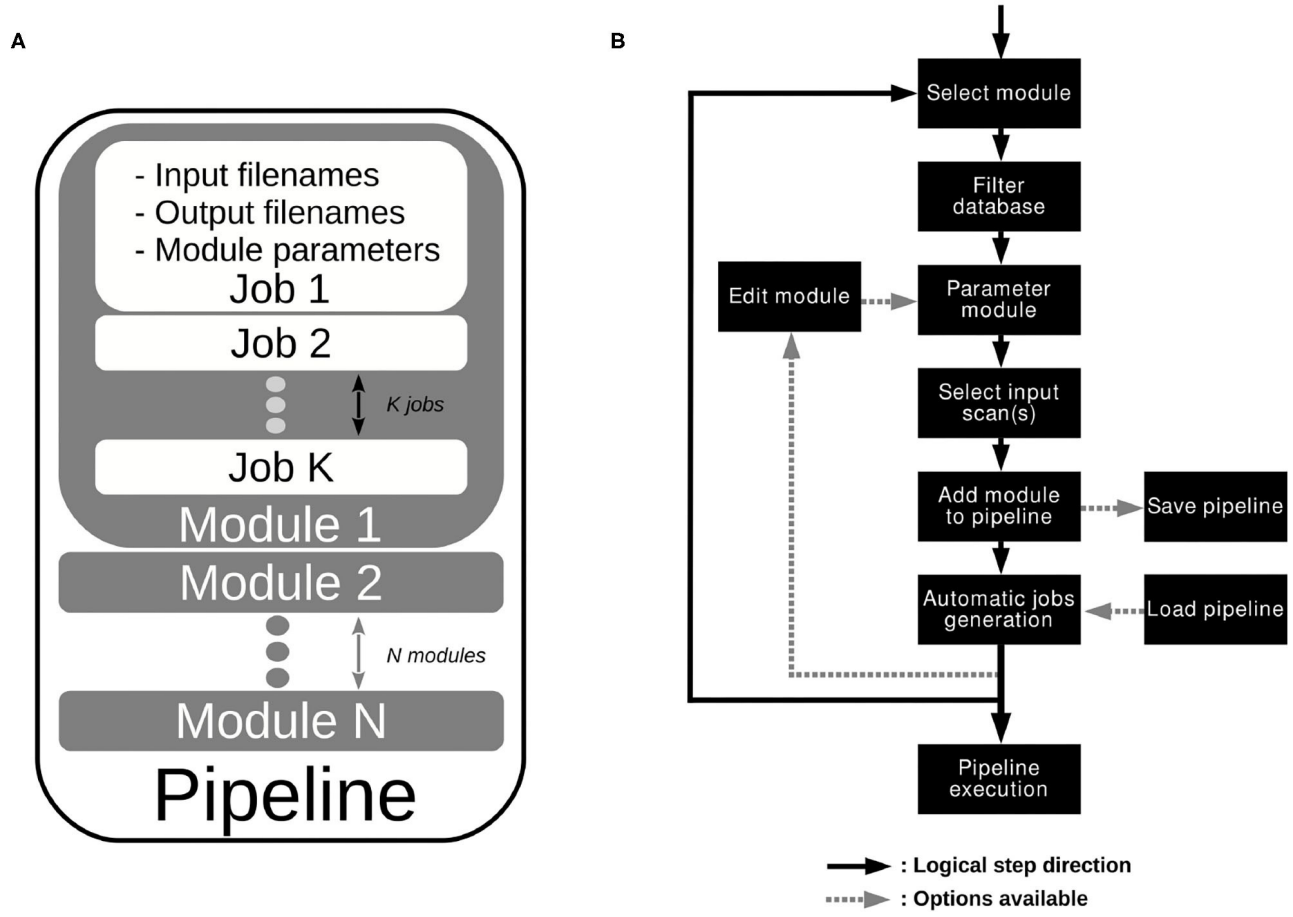
**(A)** Pipeline architecture. **(B)** Work flow editor.

As illustrated on Figure 2A, a pipeline is composed of one or several *modules*. A module is a function, more or less complex, that is designed to compute one or more NIfTI files thanks to one or more other NIfTI files, and some parameters. For instance, a module called *Module_Smooth* takes as input a Sequence_Name-Tag and 3 parameters (the dimension of the filter—2D or 3D Gaussian, the size of the filter—the variance of the Gaussian, and the Extension string of the output files), and generates a smoothed image. A module shall contain a basic operation, although nothing prevent a module from containing a whole process. A module applied to a database creates a certain number of *jobs*, i.e., a certain number of occurrences of that module. Therefore, since a pipeline is simply a sequence of modules linked together, applying a pipeline to a database creates a certain number of jobs for each module. This is potentially a large number of jobs.

#### 2.5.3. Procedure to Create a Pipeline

Now that the concept of pipeline is introduced, we share the procedure to create one (Figure 2B). First, one needs to select the part of the data on which to execute the pipeline. Indeed, MP3 allows the user to apply a pipeline to the entire database or only on a sub-part of it (i.e., the 10 first subjects, or on a specific timepoint). This operation is managed by the “database filter” on the upper left corner (Figure 1D). This part of the GUI contains manual or predefined filters as well as a table that shows the filtered database. After the data selection, one can go to the module creator (Figure 1D) and choose a module among all the ones currently available (section 2.6.1). Selecting a module displays all its required parameters. For instance, selecting the *Module_Smooth* shows a line aimed to select a Sequence_Name-Tag value, another aimed to set a filter size, etc. After the modification of all parameters (which can also be left to their default value), one can add the module to the pipeline by clicking on the button *add module to a pipeline*. When one adds a module to a pipeline, the module automatically creates as many jobs as necessary (depending on the database). Each output Scan or ROI of each job is then added to the filtered database displayed on the upper left. Since the pipeline has not been executed yet, all those files do not exist yet but are already accessible for the user in the displayed database. This offers the possibility to parameterize another module with the tags of those *virtual entries*, and therefore link some modules together. The operation of filtering the database, selecting a module, parameterizing it, and adding it to the pipeline, which creates jobs (Figure 2B), can be reproduced an unlimited number of times. The *Pipeline explorer*, on the upper right corner (Figure 1D), allows an explanation of any part of the pipeline, from the different modules to each related job, to each job parameter in the job. Any operation (e.g., editing a module, deleting a module or job...) is entirely under the control of the user.

#### 2.5.4. Saving and Sharing

In order to share a pipeline between several users, computers or projects, MP3 offers the option to save and load pipelines. Since a pipeline is a list of modules, we just store in a .mat file the sequence of modules with their parameters, but we do not store the jobs, since they are specific to a database. Loading a pipeline consists of applying each module to the new database. If the tag values of the pipeline to load are not consistent with the database, a module cannot generate the jobs. The module name is then displayed in red in the pipeline explorer and one just needs to edit the affected module, select the adapted tag value and save it to make it compliant, which turns the entry from red to green. This color convention intends to make clear the content of a pipeline as well as the consequences of its execution. If some modules are displayed in orange, it means that some of their jobs (in orange too) have already been executed (during a previous execution of the pipeline). In that case, one can decide to overwrite these files or to delete the related jobs when executing the pipeline.

#### 2.5.5. Execution of a Pipeline

Once a pipeline is well-designed, there are two execution ways in MP3. The first way is the *Single Processing*. Each job is executed in the module order: all the jobs of the first module, then all the jobs of the second, etc. This quick execution, without strong dependencies between the modules and a low computing time, is especially useful when testing or developing a module. The second way is to use a pipeline execution system called “PSOM” (v2.2.2) (Bellec et al., 2012) by selecting the option *Multi Processing*. This powerful system enables the execution of a pipeline on several cores. The dependencies between jobs are taken into account and the jobs are then distributed upon each core. This system also offers a garbage collector in order to save Random Access Memory and a way to monitor the number of jobs launched in parallel. As it has an influence on the computer's behavior during the execution of the pipeline, one can set this number of *Workers* before the execution. During the execution, all output files are written in a temporary folder. At the end, each output file of a successful job is saved in its data folder according to its type (Derived_data or ROI_data). In order to help navigating in the database (cf. *Database filter* section), we defined a color code based on each scan type. The blue represents raw data, derived data are in green, while the pink means ROI data, and the yellow colorizes the virtual files (output files of not executed pipeline yet).

### 2.6. Modules

The power of the pipeline manager lies in its modules. They are the basic operations needed to design complex pipelines. We adapted the way to define modules exposed in PSOM (v2.2.2) (Bellec et al., 2012). A module is then a MATLAB function, stored in a .m file whose name begins by the string “*Module_*.” All those functions lie in a folder called *Modules* of the MP3 source code and are sorted by area, with all the modules of an area in a folder. On launch, the pipeline manager reads the *Module* folder and its sub folders to list every available module. Thereby, the addition or deletion of a file in the subfolders of this repository updates the module list when launching the pipeline manager.

#### 2.6.1. Provided Modules

MP3 is provided along with 12 modules, performing basic operations, such as smooth, threshold, or mask an image with a ROI, compute a brain extraction module (Chou et al., 2011), export a .csv file containing first order statistics or an HTML report of images and ROI. We also provide modules used in the preclinical pipeline exposed section 3.1, which are MRI oriented, and a module able to delete files of the project's database. Finally, a module interfacing the famous toolbox SPM (Software, 2020) allows reslicing images to match the referential of a reference image.

#### 2.6.2. Template

Other users must be able to create their own modules. To facilitate the development of a new module, a module named *Module_Template* is available. It is extensively commented and explains how a module works. One then has to complete and adapt it to the wanted behavior and to save the new .m file in the *Module* folder to rapidly apply a new module on a database. To create a new module, even if it is simply an interface with another language, one need to know the basics of MATLAB programming.

#### 2.6.3. Traceability

At each job execution, the module parameters and all the input/output filenames are stored in the JSON file associated with each output scan. Thereby, a scan obtained through the pipeline manager is linked to the raw data through the history of each module applied to the raw scan, and each module parameters. A basic GUI, called “File History,” launchable from the viewer when a scan is loaded, is able to display all of this history. For each scan, we can go back to the past and know exactly how this scan was obtained and when each module has been executed.

#### 2.6.4. Module Repository

The homemade modules are aimed to gather the laboratory knowledge and know how. It is also a way to avoid loss of skill due to the departure of a team member. For example, the development team wrote more than 60 modules that match our needs of image processing. All those modules are available at https://github.com/nifm-gin/MP3_User_Modules_Repository. One can find MRI oriented modules, interfaces to others toolboxes functions, such as the co-registration modules from Software (2020) or Jenkinson et al. (2012), or more advanced processing, such as clustering, texture analysis, MR fingerprinting analysis, quantitative MR relaxometry, MR perfusion analysis (cerebral blood flow, cerebral blood volume, vessels size, vessels permeability, etc.).

## 3. Results

MP3 has been verified and validated on several MRI and CT data studies, but nothing prevents its use on any file that can be converted in NIfTI.

### 3.1. Preclinical Application

**Figure 3:**
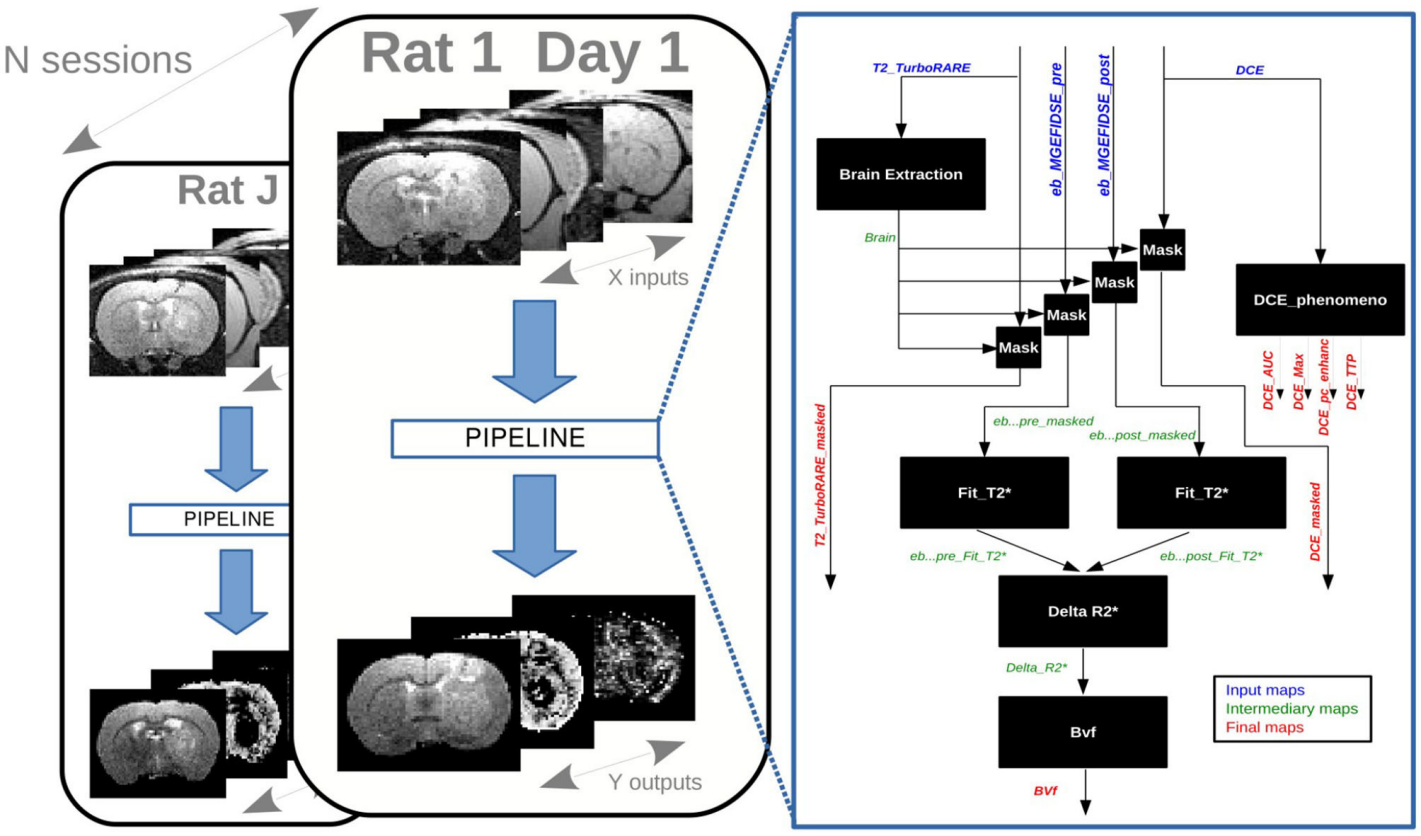
Preclinical study: occurrences of a pipeline on a database.

A research study published in 2017 compared the blood-barrier permeability changes induced by synchrotron microbeam or uniform radiation therapy. Eighteen rats bearing intracranial tumors were treated and imaged by multi-modal MRI using the Grenoble MRI facility IRMaGE (Bouchet et al., 2017). All procedures related to animal care conformed to the Guidelines of the French Government with licenses 380325 and 380321 (authorized lab A3818510002 and A3851610004). Part of these data are available online as an example of a MP3 project (Dataset, 2019). This project contains data of two rats among the 18 ones each imaged at three timepoints. Each timepoint contains 10 scans corresponding to the data described in Bouchet et al. (2017), a ROI delineating the brain, and some masked scans obtained thanks to the former ROI. The data processing, composed of 16 modules, was designed as an MP3 pipeline and applied on the database of the study. As shown on Figure 3, it created several occurrences of the pipeline aimed to process the data of each timepoint of each subject in the same way.

### 3.2. Clinical Application

**Figure 4:**
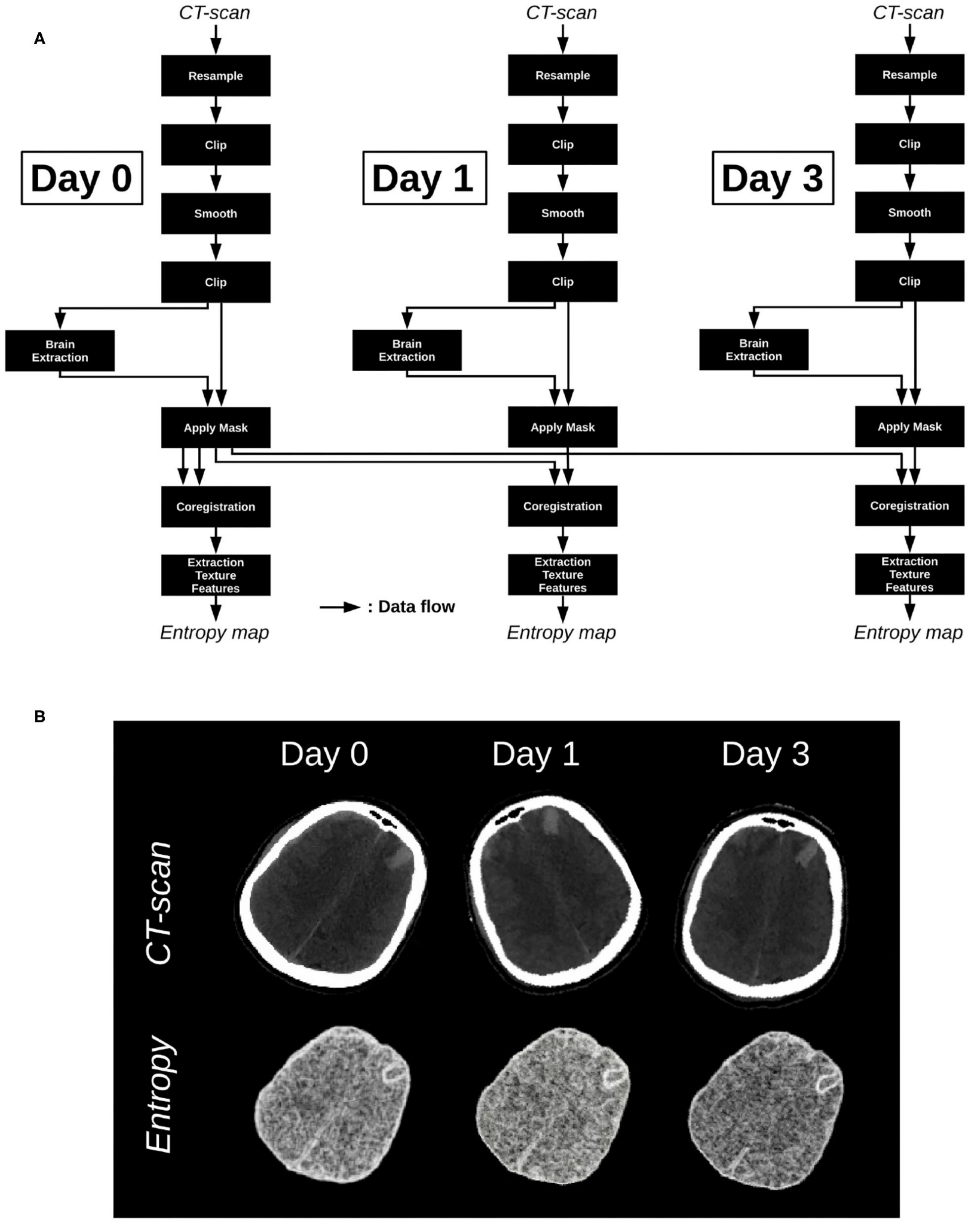
**(A)** Processing pipeline applied on the images acquired at day 0, day 1, and day 3. **(B)** Input maps: CT-scans, and output maps: Entropy maps.

As previously said, any image able to be converted in Bruker, DICOM, PAR/REC, or NIfTI format can be processed in MP3, no matter its nature. For instance, MP3 is used in a non-published study aimed to predict the evolution of brain injuries following a traumatic brain injury (TBI) using CT-scans acquired at the admission in the hospital, and at respectively 1 and 3 days after. A pre-processing pipeline for CT-scans, defined and described by Muschelli et al. (2015) was integrated as part of our own post-processing pipeline. This process resizes, clips, filters and extracts the brain from CT-scans. For our study, we added a module of coregistration (between the timepoints of a patient) from the SPM toolbox (Software, 2020) and a homemade module able to process a local entropy map, as well as some other texture images. All those modules are available at https://github.com/nifm-gin/MP3_User_Modules_Repository. The pipeline applied to a patient and its three timepoints is described on Figure 4A. This patient is part of a cohort whose data acquisition was allowed by the French institution “Comité de protection des personnes” and respects the patients written inform consent obligation. The CT-scans and entropy maps, respectively inputs and outputs of the pipeline, are displayed on Figure 4B. One can see that entropy scans are co-registered and skulls were well-removed. Such pipeline may be easily applied to a large sample of patients in order to follow the evolution of the brain entropy, used as a potential prognostic biomarker of neurological outcome.

## 4. Limitations

Despite all the work done to make it accessible and useful to the needs of the greatest number of people, MP3 does not yet offer all the performance that can be found in the best software in research fields such as physics or astronomy, which are more advanced on these aspects of interoperability and data reuse. MP3 does not meet all the FAIR (Findable, Accessible, Interoperable, Reusable) principles, which aim at improving the sharing of digital resources as described by Wilkinson et al. (2016). Indeed, MP3 is not based on a real database that is easily adaptable, nor on a standardized data architecture. Since standards definition in the medical imaging field was only at its beginning when we started the development of MP3 we did not integrate them deeply in the software. However, we decided to make MP3 compatible with recently defined standards, such as the BIDS convention (through our import/export functions). Another limitation concerns the execution of jobs on an external cluster that is not available today. Although this is conceptually possible, it would require additional development and testing. However, a multi-core execution is easy to use from a local server or computer. For example, we have tested MP3 on a project containing 450 subjects, 1,100 sessions for a total of 10,000 scans (3D, 4D, or even 5D). In this project, we successfully executed (on 32 CPU in parallel), onto the entire database a complex processing pipeline of 18 jobs per session which represented around 20,000 jobs.

## 5. Conclusion

This article exposed a new open source software able to support an end-to-end research study on a large amount of data. Thanks to three graphical interfaces, MP3 offers to convert and visualize medical images and to interact with them by comparing or analyzing them. Based on a pipeline execution system called PSOM, MP3 also enables the creation, management, and execution of complex pipelines on heterogeneous cohorts described in databases, which handle time dependencies and multiparametric data. MP3 can be used either by end-used (non-developers) or by developers which can improve it or develop their own modules. MP3 has been tested on several studies, from preclinical to clinical, from MRI to CT data, and allowed us to process a preclinical cohort of more than 450 animals. MP3 can be downloaded on github: https://github.com/nifm-gin/MP3 and modules developed by our lab (more than 60) are available on: https://github.com/nifm-gin/MP3_User_Modules_Repository.

To conclude, we believe that, despite its limitations, MP3 makes it possible to facilitate large cohort analysis (preclinical/clinical) while improving the robustness and reproducibility of medical imaging studies. Indeed, one can think that in a near future every scientific publication will have to make both the raw data but also the processing pipeline used available and software such as MP3 will be able to facilitate this step.

## 6. Requirements

MP3 can be run on MATLAB 2017b and higher and needs the Image Processing Toolbox. In order to fully enjoy the software, we recommend installing the Statistics and Machine Learning Toolbox and the Parallel Computing Toolbox. Since the converter GUI is developed in Java, Java 8 is also required. Any of the three main Operating System (OS) can handle this software. MP3 is open source, open development and available on github (Software, 2019). To develop new modules, one needs to know MATLAB programming.

## Data Availability Statement

The datasets presented in this study can be found in online repositories. The names of the repository/repositories and accession number(s) can be found at: https://github.com/nifm-gin/MP3; https://github.com/nifm-gin/MP3_User_Modules_Repository.

## Ethics Statement

The study involving human participants were reviewed and approved by the French institution Comité de protection des personnes. The patients/participants provided their written informed consent to participate in this study. The animal study was reviewed and approved by the Guidelines of the French Government with licenses 380325 and 380321 (authorized lab A3818510002 and A3851610004). Written informed consent was obtained from the individual(s) for the publication of any potentially identifiable images or data included in this article.

## Author Contributions

All authors listed have made a substantial, direct and intellectual contribution to the work, approved it for publication, proofread, and corrected the final manuscript. CB and BL co-developed the main software. OM developed the converter. CB, FB, AD, and BL co-developed modules for MP3. CB and BL wrote the first draft of the paper.

## Conflict of Interest

The authors declare that the research was conducted in the absence of any commercial or financial relationships that could be construed as a potential conflict of interest.
